# A mixed method feasibility study of a patient- and family-centred advance care planning intervention for cancer patients

**DOI:** 10.1186/s12904-015-0023-1

**Published:** 2015-05-16

**Authors:** Natasha Michael, Clare O’Callaghan, Angela Baird, Karla Gough, Mei Krishnasamy, Nathaniel Hiscock, Josephine Clayton

**Affiliations:** 1 Palliative Care Service, Cabrini Health, 646 High Street, Prahran, Victoria, 3181 Australia; 2 Faculty of Medicine, Nursing and Health Sciences, Monash University, Victoria, 3800 Australia; 3 Caritas Christi Hospice, St Vincent’s Hospital, 104 Studley Park Rd Kew, Victoria, 3101 Australia; 4 Department of Medicine, St Vincent’s Hospital, Medicine, Dentistry and Health Sciences, The University of Melbourne, Parkville, Victoria, 3010 Australia; 5 Peter MacCallum Cancer Centre, St Andrews Place, East Melbourne, VIC 3002 Australia; 6 Department of Nursing, University of Melbourne, Carlton, VIC 3053 Australia; 7 HammondCare Palliative and Supportive Care Service, Greenwich Hospital, 97-115 River Road, Greenwich, NSW 2065 Australia; 8 Sydney Medical School, University of Sydney, New South Wales, 2006 Australia

**Keywords:** Advanced care planning, Palliative care, Patients, Caregivers, Cancer, Mixed methods research

## Abstract

**Background:**

Advance care planning (ACP) is a process whereby values and goals are sensitively explored and documented to uphold patients’ wishes should they become incompetent to make decisions in the future. Evidenced-based, effective approaches are needed. This study sought to assess the feasibility and acceptability of an ACP intervention informed by phase 1 findings and assessed the suitability of measures for a phase 3 trial.

**Methods:**

Prospective, longitudinal, mixed methods study with convenience sampling. A skilled facilitator conducted an ACP intervention with stage III/IV cancer patients and invited caregivers. It incorporated the vignette technique and optional completion/integration of ACP documents into electronic medical records (EMR). Quantitative and qualitative data were collected concurrently, analysed separately, and the two sets of findings converged.

**Results:**

Forty-seven percent consent rate with 30 patients and 26 caregivers completing the intervention. Ninety percent of patient participants had not or probably not written future care plans. Compliance with assessments was high and missing responses to items low. Small- to medium-sized changes were observed on a number of patients and caregiver completed measures, but confidence intervals were typically wide and most included zero. An increase in distress was reported; however, all believed the intervention should be made available. Eleven documents from nine patients were incorporated into EMR. ACP may not be furthered because of intervention inadequacies, busy lives, and reluctance to plan ahead.

**Conclusions:**

In this phase 2 study we demonstrated feasibility of recruitment and acceptability of the ACP intervention and most outcome measures. However, patient/family preferences about when and whether to document ACP components need to be respected. Thus flexibility to accommodate variability in intervention delivery, tailored to individual patient/family preferences, may be required for phase 3 research.

**Electronic supplementary material:**

The online version of this article (doi:10.1186/s12904-015-0023-1) contains supplementary material, which is available to authorized users.

## Background

Decision-making in cancer care is increasingly complex as therapeutic options increase alongside ongoing ambiguity about acceptable outcomes for patients with advanced illness. Unexpected patient deterioration may necessitate difficult conversations and ad hoc decision-making, contributing to significant patient and family distress [1]. Early conversations between patients, caregivers and health professionals are encouraged to ascertain when cancer patients may want to consider treatment limitations to avoid inappropriate and aggressive care at advanced stages of illness [2,3].

Advance care planning (ACP) is a process whereby values and goals are sensitively explored and documented to uphold patients’ wishes should they become incompetent to make decisions in the future [4]. ACP conversations have broader benefits in enhancing patient [5] and caregiver [6] confidence, encouraging involvement in health care decision-making and allowing for consideration for additional end-of-life contingencies whilst the patient is still competent. It is thus a core quality indicator in cancer care [7], intended to improve the quality of death and family bereavement experiences [8].

ACP in Australia is increasingly implemented and accepted across health and community sectors [9], with limited uptake of advance directives, variances across state legislation and a national framework [10] developed to promote uniformity in practice. However, ACP in the Australian cancer context is not routine and remains underexplored. Its association with loss of hope [11,12], oncologists’ reticence in initiating ACP conversations [13,14], variances in preferences for prognostic information [15], occasional discrepancy between patients’ and caregivers’ desire for shared end of life (EOL) discussions [6], and the iterative and dynamic nature of EOL decision-making [16] confound healthcare workers’ considerations about optimal and timely information provision in the cancer context.

Given challenges associated with EOL conversations [17,18], structured interventions [19] and decision aids [20] are encouraged to promote understanding of management options and decision-making. Decision aids for ACP in particular allow for a systemized approach to inform patients about care options, prompting them to document and communicate their preferences [20]. However, studies utilizing oral and printed information, video material, patient narratives, and case vignettes [19,21-25] have produced varying results. A systematic review of 55 studies assessing ACP interventions with older adults found that patients preferred to discuss future health care plans with family rather than healthcare providers, complete informal (35.9%) rather than formal (22.7%) plans, and advanced directive completion rates improved when professional teams provided assistance across multiple sessions [22]. Furthermore, a Cochrane review of 131 decision aids in varied clinical contexts demonstrated positive effects on decision-making. Compared to usual care, decision aids were associated with reduced decisional conflict and improved: knowledge of options and their potential benefits, harms, and outcomes; participation in decision-making; and congruence between values and choices [26].

Yet many trials of ACP interventions or decision aids omit preliminary investigation of clinical efficacy, safety, recruitment potential, and resource requirements [21,27-29]. Given limited evidence for cancer specific ACP interventions, a research project commenced in 2012 in accordance with the Medical Research Council framework for developing complex interventions [30]. The aim of this phase 2 study was to assess the feasibility and acceptability of an ACP intervention which incorporated basic principles of local [9] and international [31,32] ACP programs as well as findings from completed phase 1 studies [6,16]. It also aimed to assess feasibility of measures to be used in a phase 3 trial of the intervention.

## Method

### Design

**Figure 1:**
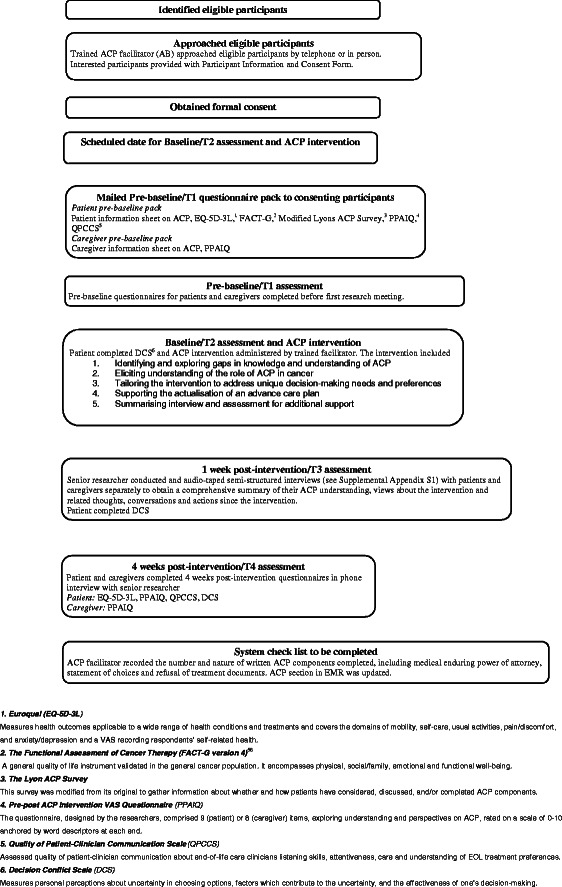
Study schema [56].

A prospective longitudinal mixed methods study with convenience sampling was used [33]. The ACP intervention was conducted by an experienced oncology nurse, with post graduate training in palliative care and many years experience conducting end-of-life conversations with cancer patients. It included a 5 step guided process as described in Figure 1 (online - Additional file 1). Development of the ACP intervention was informed by phase 1 findings on cancer patients’ and caregivers’ ACP related views and their recommendations for ACP program development [6,16]. The study included patient nominated caregiver presence, and optional completion and integration of patients’ ACP documents into the hospital’s electronic medical records (EMR). ACP documents which could be offered included an Enduring Power of Attorney (Medical Treatment) (EPOAMT) form, a researcher created ‘statement of choices’ form, and Refusal of Treatment Certificate. An EPOAMT is the Victorian (state of Australia) term to denote one’s designated and lawful substitute medical decision maker and a ‘statement of choices’ is the Australian term for documents where people state their wishes to assist substitute decision makers and doctors making decisions on their behalf. A Refusal of Treatment Certificate allows Victorian people to “legally refuse treatment generally or of a particular kind for a current condition” [9]. It also incorporated four clinical case vignettes, developed and tested in Phase 1 to initiate and support ACP discussions [6,16]. The clinical vignettes were used within the intervention to highlight circumstances where an ACP could assist families and professionals with appropriate end-of-life conversations and patient care decisions. Vignettes are useful when exploring sensitive issues [34] to assist participants reflect from a less threatening third person perspective and to introduce personal experiences when desired [35].

### Participants and setting

This study was conducted at a large specialist oncology facility in Australia. Participants were recruited from the lung, gastrointestinal, sarcoma, head and neck and urological streams between February and July 2013. Ethics approval was obtained from the Peter MacCallum Cancer Centre’s Human Research Ethics Committee.

Recruitment was limited to English speaking cancer patients and caregivers 18 years or older, previously unknown to the intervention facilitator and research interviewer, and had not participated in the phase 1 studies. Patients also had stage III/IV disease, prognosis of > 6 weeks, and Australian Karnofsky Performance status > 40.

### Study procedure

The study procedure, measures used, and brief description of the intervention are in Figure 1. A detailed description of the ACP intervention is available online (Additional file 1).

### Sample size

The pragmatic sample size of thirty patient-caregiver dyads was expected based on Phase 1 recruitment [6,16] and funds available.

### Analysis

Quantitative and qualitative data were collected concurrently, analysed separately, and the two sets of findings converged [36].

#### Statistical analysis

All quantitative analysis was performed through SPSS Windows Version 21.0 [37]. Prior to formal analysis, descriptive statistics and graphical displays were used to identify missing values and to examine the data distribution. Descriptive statistics were also used to summarise characteristics relevant to participant flow, compliance with assessments and questionnaires, pre-baseline participant characteristics (patients and caregivers) and responses to study measures.

For the Euroqual-5D (EQ-5D-3L) [38] and Pre-post ACP Intervention Questionnaire (PPAIQ), paired-samples t-tests were used to calculate estimates of change at T4 from T1 with 95% confidence intervals. Analysis of the Decisional Conflict Scale (DCS) [39] was carried out by fitting a linear mixed model to all available data. A reference cell model was used to estimate mean changes from T2 at follow-up assessment with 95% confidence intervals [40]. The mixed model was estimated by maximum likelihood and an unstructured covariance type was used to model the covariance structure among repeated measures. Effect size (ES) estimates were calculated to characterise the size of before and after changes [41]; these were interpreted as per Cohen’s *d* (0.2, small; 0.5, medium; and 0.8, large change) [42].

#### Qualitative analysis

Analytic strategies were derived from grounded theory methods [43]. Patient and caregiver transcribed interviews underwent inductive, cyclic and constant comparative analysis. Initially, patient and caregiver data were separately coded and comparable codes grouped into sub-categories. Patient and caregiver sub-categories were then compared and grouped into categories. Comparable categories were grouped into themes. Qualitative data management software was used [44]. Initial analysis was conducted by CO with subsequent inter-rater reliability [45] provided by NM and AB.

## Results

### Trial profile

**Figure 2:**
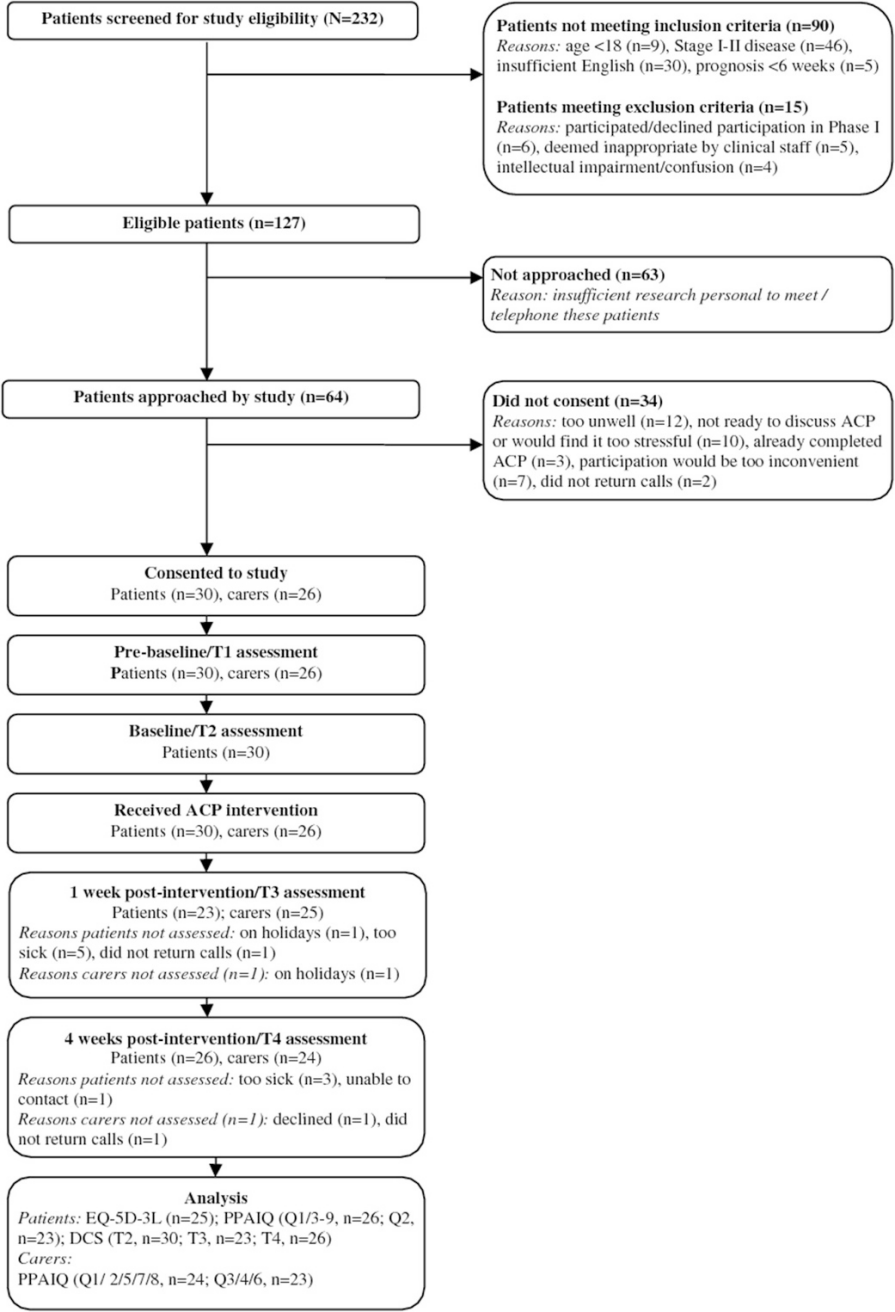
CONSORT participant flow.

Of 127 patients eligible for the study (Figure 2), 64 were approached and 30 consented (47% consent rate) between February and July 2013. Insufficient research personnel resulted in 63 eligible patients not being approached. Twenty-six caregivers also consented. One patient did not have a caregiver, one caregiver declined, and two could not attend the intervention. Patient and caregiver baseline characteristics are summarised in Table 1.

**Table 1:** **Participant characteristics**

	Patients		Carers	
	n	%	n	%
Age				
M	62.4		57.7	
SD	11.3		12.4	
Sex				
Male	19	63	8	31
Female	11	37	18	69
Marital status				
Single	3	10	4	15
Married	19	63	15	58
Defacto/living together	5	17	5	19
Separated/divorced	1	3		
Widowed	2	7	2	8
Place of birth				
Australia	20	67	19	73
Not Australia^a^	10	33	7	27
Time in Australia, if not born in Australia				
Mdn	36.5		39.0	
IQR	[22.3, 46.3]		[14.0, 50.0]	
Preferred language				
English	29	97	26	100
Other	1	3		
Highest level of education				
Less than high school	5	17	9	35
High school graduate	6	20	8	31
TAFE/University degree	13	43	6	23
Post-graduate degree	6	20	3	12
FACT-G^b^				
Physical wellbeing	17.8 (5.5)			
Social wellbeing	21.0 (4.3)			
Emotional wellbeing	16.6 (4.9)			
Functional wellbeing	16.0 (5.1)			
Total score	71.3 (15.4)			
Relationship to patient				
Spouse/partner			20	77
Parent			2	8
Child			3	12
Other			1	4
Length of relationship (in years)				
Mdn			38	
IQR			[23.5,45.5]	
Have you had ACP discussion with this patient?				
No			16	62
Yes			10	38

^a^Not Australia includes England (2 patients, 3 carers), Greece (1 patient), Hong Kong (1 patient, 1 carer), Ireland (1 patient), Italy (1 patient), Malta (1 patient, 1 carer), New Zealand (2 patients, 1 carer), Scotland (1 patient) and Sweden (1 carer).

^b^Estimates for FACT-G are means (and standard deviations).

### Need for intervention

Modified Lyon ACP [46] survey responses assessed at baseline are summarised in Table 2. Seventy percent had not written and 20% had probably not written about future health plans. Few (7%) had heard about and completed an ACP. At least 67% had not completed an EPOAMT and had never discussed their wishes for care at the EOL.

**Table 2:** **Modified Lyons ACP survey responses**

Item	n	%
Have you ever written down any thoughts about your future health plans?		
Yes, definitely	1	3.3
Very probably	1	3.3
Probably	1	3.3
Probably not	6	20.0
Definite no	21	70.0
Don't know		
Advance Care Plans allow people to make their health care choices known before becoming seriously ill.		
Have heard about and completed	2	6.7
Have heard about but not completed	13	43.3
Have not heard about	13	43.3
Don't know	2	6.7
Have you ever heard about and completed a Medical Enduring Power of Attorney in which you name someone to make decisions about your health care in case you could not?		
Have heard about and completed	8	26.7
Have heard about but not completed	14	46.7
Have not heard about	6	20.0
Don't know	2	6.7
Whether you have completed any Advance Care Planning or not, have you talked about your wishes for care at the end of life with anyone?		
Spouse/partner	13	44.8
Parents	1	3.4
Siblings	9	31.0
Friends	2	6.9
Boyfriend/girlfriend	1	3.4
Primary physician	1	3.4
Clergy	1	3.4
Other	7	24.1
Have not talked with anyone	12	41.4

### Intervention fidelity and characteristics

All 30 patients completed the ACP intervention, including 26 with nominated caregivers. Seventeen interventions were completed at the specialist oncology facility (9 outpatients, 5 inpatients, 3 in day chemotherapy unit) and 13 at patients’ homes. Mean ACP intervention completion time was 44 (SD 9) minutes.

### Compliance with assessments and questionnaires

Apart from 1 week post-intervention/T3 (77%), compliance with assessments was high for patients (≥87%) and caregivers (≥92%). Missing responses to items comprising each questionnaire was very low for patients (<2.5%) and caregivers (<1.0%).

### Estimate of change

Descriptives for study measures EQ-5D-3L and DCS, completed by patients at pre-baseline, baseline and follow-up assessments, are in Table 3. Descriptives were not calculated for the Quality of Patient-Clinician Communication Scale (QPCCS) [47] due to the high rate of missing data. Descriptives for study measures completed by caregivers are available from the authors.

**Table 3:** **Descriptives for study measures completed by patients at pre-baseline, baseline and follow-ups**

Study measure	Assessment											
	Pre-baseline			Baseline			1 week post-intervention			4 weeks post-intervention		
	n	M	SD	n	M	SD	n	M	SD	n	M	SD
EQ-5D												
Visual Analogue Scale (0=worst imaginable to 100=best imaginable health state)	29	60.6	19.1							26	69.9	20.0
Pre-post ACP Intervention Questionnaire												
1. I rate my current understanding of ACP as (0=poorest to 10=best possible understanding)	30	6.5	2.4							26	7.6	1.8
2. The opportunity to consider my possible future health care needs and wishes with health care professionals have been as (0=unsatisfying to 10=satisfying as possible)	27	6.8	2.2							25	8.0	1.5
3. Thinking about my possible future health care needs and wishes if I became unwell causes me (0=lowest to 10=highest distress imaginable)	30	3.4	2.2							26	4.6	2.7
4. Discussing my possible future care health needs and wishes with others would cause me the (0=lowest to 10=highest distress imaginable)	30	3.6	2.6							26	3.8	2.5
5. Making and informing others about decisions related to my possible future health care needs and wishes is (0=not important at all to 10=extremely important)	30	7.8	1.8							26	8.4	1.9
6. My confidence in discussion of my possible future health care needs and wishes with health professionals is (0=lowest to 10=highest possible)	30	7.7	2.2							26	8.4	1.3
7. My confidence in discussion of my possible future health care needs and wishes with family members/friends is (0=lowest to 10=highest possible)	30	8.0	2.2							26	8.4	1.7
8. Considering my advanced care plan is (0=not helpful at all to 10=extremely helpful)	30	7.8	2.0							26	8.0	2.7
9. Considering advance care planning when living with a cancer diagnosis is (0=not important at all to 10=always important)	30	8.0	1.9							26	8.6	2.3
Decision Conflict Scale												
Total score (0=no to 100=extremely high decisional conflict)				30	23.9	16.5	22	22.0	15.3	26	19.7	15.9
Uncertainty subscore (0=extremely certain to 100=extremely uncertain about best choice)				30	28.9	23.6	23	27.2	21.6	26	22.4	21.4
Informed subscore (0=feels extremely informed to 100=feels extremely uninformed)				30	22.2	17.4	22	24.2	23.6	26	19.9	16.8
Values clarity subscore (0=feels extremely clear to 100=feels extremely unclear about personal values for benefits & risks/side effects)				30	29.4	19.4	22	27.7	22.9	26	22.1	16.7
Support subscore (0=feels extremely support to 100=feels extremely unsupported in decision making)				30	20.3	16.2	22	14.4	12.4	26	15.7	15.5
Effective decision subscore (0=good decision to 100=bad decision)				30	20.0	17.4	23	18.8	11.8	26	18.5	17.9

Estimates of change and effect size for study measures completed by patients only (EQ-5D-3L, DCS) and by patients and caregivers (PPAIQ) are in Table 4.

**Table 4:** **Estimates of change and effect size for study measures completed patients (EQ-5D-3L, DCS and PPAIQ) and carers (PPAIQ)**

EQ-5D-3L (patients only)	M chg T1 to T4 (95% CI)	ES		
Visual Analogue Scale	6.1 (-.1, 12.3)	0.36		
Decision Conflict Scale (patients only)	M chg T2 to T3 (95% CI)	ES	M chg T2 to T4 (95% CI)	ES
Total score	-1.2 (-7.1, 4.8)	0.07	-4.7 (-11.6, 2.2)	0.29
Uncertainty subscore	-2.5 (-11.7, 6.6)	0.11	-7.1 (-16.3, 2.2)	0.31
Informed subscore	3.4 (-4.4, 11.3)	0.2	-2.5 (-11.1, 6.1)	0.15
Values clarity subscore	-1.0 (-11.6, 9.6)	0.05	-7.4 (-16.8, 1.9)	0.39
Support subscore	-5.2 (-10.4, 0.03)	0.33	-5.1 (-11.3, 1.0)	0.32
Effective decision subscore	-1.2 (-6.2, 3.8)	0.07	-1.9 (-8.8, 4.9)	0.11
	Patients		Carers	
Pre-post ACP Intervention Questionnaire (patients and carers)	M chg T1 to T4 (95% CI)	ES	M chg T1 to T4 (95% CI)	ES
1. I rate my current understanding of ACP as …	1.2 (-0.02, 2.3)	0.50	1.0 (0.1, 1.9)	0.40
2. The opportunity to consider my possible future health care needs and wishes with health care professionals have been as …	1.1 (-0.01, 2.2)	0.58	1.9 (0.8, 2.9)	0.83
3. Thinking about my possible future health care needs and wishes if I became unwell causes me …	1.1 (0.2, 2.0)	0.50	.5 (-0.7, 1.7)	0.19
4. Discussing my possible future care health needs and wishes with others would cause me the …	0.3 (-0.4, 1.1)	0.14	.5 (-0.8, 1.9)	0.21
5. Making and informing others about decisions related to my possible future health care needs and wishes is …	0.6 (-0.3, 1.4)	0.30	.2 (-0.3, 0.6)	0.19
6. My confidence in discussion of my possible future health care needs and wishes with health professionals is …	0.4 (-0.3, 1.2)	0.21	1.6 (0.2, 3.0)	0.56
7. My confidence in discussion of my possible future health care needs and wishes with family members/friends is …	0.4 (-0.1, 0.9)	0.19	0.5 (-0.3, 1.4)	0.33
8. Considering my advanced care plan is …	0.2 (-0.9, 1.2)	0.08	-0.08 (-0.6, 0.5)	0.06
9. Considering advance care planning when living with a cancer diagnosis is …	0.5 (-0.04, 1.0)	0.25	1.0 (0.1, 1.9)	0.40

M chg: mean change, T1: pre-baseline, T2: baseline, T3: 1 week post-intervention, T4: 4 weeks post-intervention, ES: effect size.

#### a) Decision conflict scale (DCS)

For patients, an improvement of 4.7 points (95% CI: −11.6, 2.2; ES = 0.29) was observed on the DCS Total score, indicating lower decisional conflict at 4 weeks post-intervention. Taking into consideration the rescaling of summed responses to items comprising the DCS Total score, this is the equivalent of about a 1-point change of improvement on any three DCS items (or, alternatively, a 3-point change of improvement on one item). In terms of DCS sub scores, the magnitude of change was greatest for values clarity (ES = 0.39), support (ES = 0.32) and uncertainty (ES = 0.31), reflecting small-sized improvements in clarity, perceived support in decision-making, and certainty about a better choice being made.

#### b) Pre-post ACP intervention questionnaire (PPAIQ)

Patients’ responses to questions 1 and 2 of the PPAIQ indicated medium-sized improvements of 1.2 (95% CI: −0.02, 2.3; ES = 0.50) and 1.1 (95% CI: −0.01, 2.2; ES = 0.58) points by 4 weeks post-intervention in understanding of ACP and satisfaction associated with opportunities to consider possible future health care needs and wishes with health care professionals. An increase of 1.1 points (95% CI: 0.2, 2.0; ES = 0.50) was also observed on question 3 at the last follow-up, indicating a medium-sized increase in patients’ level of distress associated with thinking about possible future health care needs and wishes if they became unwell.

Caregivers’ responses to question 2 indicated a large-sized improvement of 1.9 points (95% CI: 0.8, 2.9; ES = 0.83) by 4 weeks post-intervention in satisfaction associated with opportunities to consider possible future health care needs and wishes with health care professionals. A medium-sized improvement of 1.6 points (95% CI: 0.2, 3.0; ES = 0.56) was also observed on question 6, indicating greater confidence in discussion of possible health care needs and wishes with health professionals.

### Integration of ACP into EMR

Thirty patients were offered ACP documents to complete, 28 accepted them. Participants were invited to return previously or recently completed documents for scanning into the EMR. Eleven documents from 9 patients were returned which included 6 statements of choices and 5 EPOAMT.

### Participation in follow up interview 1 week post-intervention

Most patients (23/30) and caregivers (25/26) participated in the 1 week post-intervention semi-structured interview. Non-participation reasons were: unable to contact (2 patients; 1 caregiver); too unwell (5 patients). Mean patient interview time was 22 (SD 9) minutes and mean caregiver interview time was 21 (SD 9) minutes.

Three themes addressing feasibility and acceptability were identified:

### An ACP intervention may motivate participants to consider, actualise, or alter existing ACP’s

The ACP intervention initiated or extended many patients’ thoughts and discussions about their future health care desires should they lose their ability to state their views in the future. These discussions could be conducted with caregivers, other family members, and health professionals. Some caregivers especially welcomed becoming more aware of patients’ EOL care desires. One recently married 42-year-old caregiver was shocked to hear her husband’s preference to die in hospital because the patient remained distressed by memories of caring for his first wife who died from cancer and he did not want her “completely drained” by care-giving. Following the intervention she told him, “‘I made a promise that I would look after you’ …, he did hug me and say, ‘I know but I also know how awful it can be’, … it’s unfinished business.”

### Patients’ and caregivers’ can find the acceptable intervention reassuring, supportive, confronting or disempowering

Many participants said that the intervention helped them to feel respected, heard, valued, empowered, and relieved. A 69-year-old husband caregiver said that ACP, “lifts a huge burden from a caregiver”. Important information sharing could also result. A 38-year-old daughter added that her father had only shown “bravado” since his illness whereas after the “really helpful” intervention, “He finally talked about, you know, being worried about leaving his kids,… not being around to see his grandchildren, … prognosis.”

Some found the intervention both informative and distressing. One couple first considered that the patient may not recover during the intervention. A 64-year-old patient said, “It puts you on a dead end, … it was confronting but on the other hand, just, you have to make a decision.”

Occasional participants feared ACP implied that patients were close to death. A 65-year-old patient “physically had to be sick” after the intervention, adding, “It really knocked me to think that I’m being put in that pigeon hole. I mean I want to live …. It’s bad enough me having to retire.” However, this patient thought that ACP should be an available option, as did all study participants.

Patients and caregivers usually welcomed partaking in the intervention together, with many patients stating that they would not complete it alone. It occasionally, however, exposed family tension. One 68-year-old caregiver felt that her attitudes remained disrespected following the intervention, adding that conversations with her husband are, “always punctuated with what he wants”. Another 52-year-old male patient reflected on challenges of potential caregiver role reversal with his mother. Although he thought she, “would have a pretty good idea of what I would want anyway without it being put down” his mother expressed concern that he, “doesn’t talk to me” and that his ACP related affairs were “left in limbo”.

### ACP components may not be furthered post interventions

The reasons for why the majority of patients did not complete any written components of ACPs following the intervention were variable. These included patients and caregivers: not feeling ready or interested in ACP; considering their exiting plans as sufficient; and believing that “you can’t plan ahead”. Many had busy lives which involved dealing with the patients’ illnesses and some found the intervention inadequate. Two participants who were also physicians wanted to convey more specific medical details about their care and considered the documents as inadequate for these purposes. A 32-year-old male said, “It was more of an introduction .... I probably wasn’t too sure about how to proceed further.”

## Discussion

The challenge of conducting research in patients with advanced illness is well established [48]. Our results however established the feasibility of an ACP intervention specifically developed for cancer patients with stage III/IV disease, demonstrating a high compliance with the intervention, assessments and questionnaires. The QPCCS [39], however, was often unanswerable because many patients had not engaged in EOL discussions with physicians. Following the intervention, participants chose the extent to which they considered, discussed, or wrote down future plans, if at all. Only 9 patients submitted ACP documents for integration into EMR during the 4 week data collection phase, reinforcing that cancer patients often need time to approach ACP and may not be suited to interventions expecting document completion, especially if required within a short time-frame. While some emphasized their disinterest in ACP, all believed that the intervention should be available. The intervention occasionally elicited preexisting tension between patient/caregiver, however, there was no causal evidence. Although participants’ regular, positive accounts of the intervention’s effect affirmed its broad acceptability, caution is needed because one participant became distressed and over half declined study involvement.

The modified Lyons [46] survey findings demonstrate poor pre-existing knowledge and actualisation of ACP. This was not unexpected given that the authors initiated this research programme to complement ongoing statewide and national initiatives to promote ACP [10,49]. In keeping with prior studies [24,50], small-sized changes, reflecting improvement, were observed in decisional conflict, with improved clarity, perceived support in decision-making, and certainty about a better choice being made observed. Nonetheless, confidence intervals for estimates of change were wide and all included zero.

Small to medium-sized changes were also observed in: patients’ understanding and satisfaction with opportunities to consider future health care needs; and caregivers’ confidence in discussions with health professionals. Again, confidence intervals were wide and most included zero. A large-sized change was observed in caregivers’ satisfaction with opportunities to consider patients’ future health care needs. Patients’ distress associated with thinking about ACPs four weeks post intervention reinforces that ACP can remain difficult for cancer patients even when its benefits are acknowledged [16].

Updated guidance for the evaluation of complex interventions [30] such as ACP promotes theoretical development and early phase piloting as we have done. We used a combination of methods: the vignette technique, a guided intervention by a skilled facilitator and a mixed methods research approach to allow for an integration of process and outcome evaluation [51]. In view of the ethical implications of including a control group in ACP intervention research with patients with advanced illnesses [52], future phase 3 studies require consideration beyond that of a standard randomized design to possibly include a stepped wedge design, high quality quasi experimental or observational study designs. Furthermore, given that phase 1 findings illustrated how ACP needs to be offered in a flexible, individualized and patient- and family-centered manner in oncology [6,16], fidelity of related interventions need to accommodate unsystematic variations in intervention delivery. For example, this may involve the incorporation of the ‘Technology Model’, which includes monitoring of the intervention, accompanying procedures manual development, training and monitoring of interventionist as well as ongoing recording of variations in intervention delivery [53].

Several issues were identified that will require revision for a larger study. We only achieved a 47% consent rate, which was significantly lower than another ACP study which examined the impact of ACP amongst elderly Australians with nonmalignant conditions [29] and oncology studies in which oncologists recruited participants [21,28], while compatible with other oncology studies with researcher or nurse recruitment [5,6,16,54]. Recruitment by medical practitioners may increase consent rates though potential participants may feel less coerced when invited by non-clinical researchers or nurses. Follow up intervals warrant reconsideration as eight cancer patients were too sick to complete follow up due to the demanding schedule of treatment and declining illness trajectory. Cancer patients with stage I-II disease should be invited to participate in future studies with opportunities to revisit ACP decisions.

There are numerous study limitations. The study is highly vulnerable to selection bias as it was conducted in a single quaternary cancer centre which treats a cohort of treatment avid cancer patients, many of which are on early phase clinical trials. A small budget limited the intervention to a single point of contact, contrary to recommendations that suggest that uptake is increased when patients have multiple interactions with staff providing information [22]. We also did not elicit reasons for the low number of ACP documents submitted for inclusion to the EMR. However, as described in previous studies, some may have chosen to reject or relinquish ACP; may have required more time to consider ACP and may prefer informal conversations over completion of formal documentation [6,16,22]. Finally, the facilitator was an experienced and confident member of the nursing team which may have positively influenced the intervention’s acceptability. A 58-year-old male patient stated, “Researcher was great to talk with, easy to talk with, she let you sit back and have your say,” and a 60-year-old female caregiver said, “She explained it really, really well, and she put me at ease, and I felt very happy to be involved in the program”.

## Conclusion

An ACP intervention for advanced cancer patients and their caregivers was developed from phase 1 data and delivered by an experienced nurse. Feasibility of recruitment and acceptability of the intervention and most outcome measures were demonstrated. Modification of the intervention, incorporation of the Technology Model and further resourcing will be required for a larger study. Although the intervention elicited many helpful EOL conversations, our studies have demonstrated that repeated invitations for participation in ACP may elicit distress in some patients and many patients continue to be reticent about completing formal ACP documentation [6,16]. We continue to advocate for the ethical principal of “respect for persons” [55] over patient autonomy in view of the distinct variability in patients’ wishes to be involved in end-of-life discussions and decision making. The results from this study support the notion that ACP should be offered to all patients with advanced cancer and tailored to their individual preferences, rather than rigidly enforced.

## Additional file

Additional file 1:
**ACP intervention.**

## Abbreviations

ACP: Advanced care planning
DCS: Decisional Conflict Scale
EMR: Electronic medical records
EPOAMT: Enduring Power of Attorney (Medical Treatment)
EOL: End of life
EQ-5D-3 L: Euroqual-5D
ES: Effect size
PPAIQ: Pre-post ACP Intervention Questionnaire
QPCCS: Quality of Patient-Clinician Communication Scale
