# Supplementary material for: A mixed method feasibility study of a patient- and family-centred advance care planning intervention for cancer patients
Source: BMC Palliat Care. 2015 May 16;14:27. doi: 10.1186/s12904-015-0023-1 (PMC4456060; doi:10.1186/s12904-015-0023-1)
Supplement: Additional file 1: — ACP intervention. [file 12904_2015_23_MOESM1_ESM.pdf]

Additional file 1: ACP intervention

1. Identifying and exploring gaps in knowledge and understanding of ACP	Facilitator (AB) clarifies understanding of ACP at the start of the intervention. The facilitator then defines ACP and its various components in simple language.
2. Eliciting understanding of the role of ACP in cancer	The facilitator presents a series of case vignettes to patients[8] and carers.[13] These elicit situations in cancer patients where an ACP may assist health professionals with decision making and overall patient care in the event that the patient loses the capacity to speak for him/herself.
3. Tailoring the intervention to address unique decision-making needs and preferences	The facilitator explores concerns that the patient or the carer may have around illness progression, goals of care and the use of life sustaining treatments. The facilitator explains the role of a medical enduring power of attorney and the process / benefits of appointing one. Patient and carers are encouraged to discuss their wishes and values that inform end of life decision-making and completing a statement of choices
4. Supporting the actualisation of an advance care plan	The patient is offered the opportunity to complete ACP documents or to have further conversations with significant others / professionals. The facilitator assists the carer in considering their willingness to support the patient in completing and subsequently enacting an ACP.
5. Summary	The facilitator summarises the interview and the patient and carer are assessed for the need for additional support form social workers / clinical psychologists or pastoral care.
